# Tolerance to Abiotic Factors of Microsclerotia and Mycelial Pellets From *Metarhizium robertsii*, and Molecular and Ultrastructural Changes During Microsclerotial Differentiation

**DOI:** 10.3389/ffunb.2021.654737

**Published:** 2021-04-30

**Authors:** Flávia R. S. Paixão, Carla Huarte-Bonnet, Cárita de S. Ribeiro-Silva, Gabriel M. Mascarin, Éverton K. K. Fernandes, Nicolás Pedrini

**Affiliations:** ^1^Instituto de Investigaciones Bioquímicas de La Plata, Centro Científico Tecnológico La Plata Consejo Nacional de Investigaciones Científicas y Técnicas–Universidad Nacional de La Plata, La Plata, Argentina; ^2^Instituto de Patologia Tropical e Saúde Pública, Universidade Federal de Goiás, Goiânia, Brazil; ^3^Laboratório de Microbiologia Ambiental, Empresa Brasileira de Pesquisa Agropecuária–Embrapa Meio Ambiente, Jaguariúna, Brazil

**Keywords:** entomopathogenic fungi, UV-B radiation, thermotolerance, oxidative stress, gene expression

## Abstract

*Metarhizium* species fungi are able to produce resistant structures termed microsclerotia, formed by compact and melanized threads of hyphae. These propagules are tolerant to desiccation and produce infective conidia; thus, they are promising candidates to use in biological control programs. In this study, we investigated the tolerance to both ultraviolet B (UV-B) radiation and heat of microsclerotia of *Metarhizium robertsii* strain ARSEF 2575. We also adapted the liquid medium and culture conditions to obtain mycelial pellets from the same isolate in order to compare these characteristics between both types of propagules. We followed the peroxisome biogenesis and studied the oxidative stress during differentiation from conidia to microsclerotia by transmission electron microscopy after staining with a peroxidase activity marker and by the expression pattern of genes potentially involved in these processes. We found that despite their twice smaller size, microsclerotia exhibited higher dry biomass, yield, and conidial productivity than mycelial pellets, both with and without UV-B and heat stresses. From the 16 genes measured, we found an induction after 96-h differentiation in the oxidative stress marker genes *MrcatA, MrcatP*, and *Mrgpx*; the peroxisome biogenesis factors *Mrpex5* and *Mrpex14/17*; and the photoprotection genes *Mrlac1* and *Mrlac2*; and *Mrlac3*. We concluded that an oxidative stress scenario is induced during microsclerotia differentiation in *M. robertsii* and confirmed that because of its tolerance to desiccation, heat, and UV-B, this fungal structure could be an excellent candidate for use in biological control of pests under tropical and subtropical climates where heat and UV radiation are detrimental to entomopathogenic fungi survival and persistence.

## Introduction

Fungal microsclerotia (from hereafter referred to as MS) are hardened masses of pigmented hyphal aggregates (50–600 μm), serving as survival structures and first described in the entomopathogenic fungi *Metarhizium* species when grown dimorphically in submerged liquid cultures. This dimorphism in *Metarhizium* lies in the transition from conidia to myceliogenic growth followed by the development of dense and compact hyphal threads forming MS. This type of overwintering fungal structure is remarkably tolerant to desiccation and capable of producing infective conidia without exogenous carbon source due to their own endogenous reserves; thus, they stand out high potential to be used as mycoinsecticide in biological control programs (Jaronski and Jackson, 2008; Jackson and Jaronski, 2009; Behle et al., 2013; Mascarin et al., 2014; Goble et al., 2016; Song et al., 2017). However, filamentous fungi can also grow as pellets by mycelial formation in submerged cultures (from hereafter referred to as P) (Nair et al., 2016; Zhang and Zhang, 2016; Veiter et al., 2018), which can be slightly distinct in morphology and probably less tolerant to environmental stresses in relation to MS.

Abiotic factors such as ultraviolet (UV) radiation and heat stress determine fungal propagule survival and persistence, and both are more pronounced in tropical and subtropical regions. These environmental factors can alter molecular structures and trigger the production of reactive oxygen species (ROS) that induce damage, reduce fungal viability, and even provoke cell death (Braga et al., 2001; Nascimento et al., 2010; Zhang et al., 2017). The UV spectrum is divided into three wavelength intervals: UV-A (315–400 nm), UV-B (280–315 nm), and UV-C (100–280 nm). The effects of UV-B tolerance are commonly reported to propagules as conidia, mycelium, or blastospores of entomopathogenic fungi (Fernandes et al., 2007, 2015; Braga et al., 2015; Rangel et al., 2015; Brancini et al., 2018; Zhao et al., 2019; Bernardo et al., 2020; Corval et al., 2021). Temperature can be also a limiting factor during morphogenesis, germination, and fungal metabolic processes (Abrashev et al., 2008; Zhang et al., 2017). In this sense, heat stress can delay and reduce the effectiveness of conidia germination, sporulation, and growth of thermosensitive entomopathogenic fungi (Fernandes et al., 2008, 2010; Paixão et al., 2019).

Cell development in microbial eukaryotes shows a correlation between ROS generation and the upregulation of specific antioxidant enzymes, such as superoxide dismutases (SODs), catalases (CATs), CAT–peroxidases, glutathione peroxidases (GPxs), and peroxiredoxins (Aguirre et al., 2005, 2006). An imbalance between ROS and antioxidant enzymes response can cause detrimental effects in cell morphology, growth rate, metabolism, and protein secretion, among others (Aguirre et al., 2006). On the other hand, ROS generation and oxidative stress are associated with sclerotia maturation in the filamentous fungi *Sclerotium rolfsii* (Georgiou et al., 2006) and *Rhizoctonia solani* (Liu et al., 2018) and with MS and MS-like propagules development in the entomopathogenic fungi *Metarhizium* species (Song et al., 2013, 2018; Liu et al., 2014) and *Beauveria bassiana* (Huarte-Bonnet et al., 2019), respectively. Peroxisome biogenesis has also been observed in *B. bassiana* during mycelial pellet formation when cultivated in liquid medium supplemented with hydrocarbons (Huarte-Bonnet et al., 2018) and during MS-like development (Huarte-Bonnet et al., 2019), accompanied by induction of oxidative stress marker genes and *pex* genes encoding for peroxins, proteins involved in the transport of matrix proteins from the cytosol into peroxisome lumen.

In this study, we grew the entomopathogenic fungus *Metarhizium robertsii* in two different culture media to obtain two fungal propagules (MS and P) with similar morphology but encompassing differences in biomass production and propagule size. As scarce information is available regarding microsclerotial tolerance to abiotic factors (Corval et al., 2021), the aims of this study were to compare the effect of UV-B radiation and heat on both MS and P tolerance and to characterize for the first time the expression pattern of genes potentially involved in oxidative stress, pigmentation, and peroxisome biogenesis during *M. robertsii* microsclerotial differentiation.

## Materials and Methods

### Production and Characterization of Microsclerotia and Mycelial Pellets From *M. robertsii*

The entomopathogenic fungus *M. robertsii* strain ARSEF 2575 was used in this study. It is deposited at the US Department of Agriculture (USDA)–ARS Collection of Entomopathogenic Fungal Cultures, Ithaca, NY. Conidia were obtained from fungal cultures grown within 10 days onto potato dextrose agar medium (Merck, Darmstadt, Germany) supplemented with 1 g L^−1^ yeast extract (Oxoid, Hampshire, England) (PDAY) in the dark at 27 ± 1°C. Ten milliliters of conidial suspensions were prepared and adjusted to 5 × 10^7^ conidia mL^−1^ with 0.05% Tween 80 [polyoxyethylene sorbitan monooleate] (Sigma–Aldrich, USA) solution, vortexed, and inoculated into Erlenmeyer flasks (250 mL), containing 90 mL of different basal media (Table 1) to produce either MS (Mascarin et al., 2014) or P (Mapari et al., 2008). Cultures were set at 27 ± 1°C in a rotary shaker incubator at 250 rpm (Certomat BS-1, Sartorius, Germany). Aliquots (1 mL) were obtained from each culture medium at different time intervals. At the last time point (96 h), the pH of the cultures was measured. In order to evaluate the accumulation of biomass, aliquots were dewatered on filter paper discs (80 g m^−2^) previously weighed. Samples were dried at 32°C for 2 days to obtain the dry biomass (Jackson and Jaronski, 2009). For evaluation of propagule concentration, 9 mL of 0.05% Tween 80 was added to 1 mL of liquid culture, and 100 μL of this suspension was placed between slides (76.2 × 25.4 mm) and coverslips (24 × 24 mm). The suspension was quantified by optical microscope at 40× magnification (Jackson and Jaronski, 2009), and propagule diameter was measured with a Leica ICC50 HD camera and Leica 201 LAZ EZ software version 3.0.0. Three tests were conducted in different days.

**Table 1:** Components of basal media used to obtain microsclerotia and pellets.

**Component**	**Molecular formula**	**Concentration (g/L)**	
		**Microsclerotia**	**Pellets**
Anhydrous dextrose	C_6_H_12_O_6_	200	20
Malt extract	—	—	20
Yeast extract	—	15	—
Casein (acid hydrolyzate)	—	—	1
Monobasic potassium phosphate	KH_2_PO_4_	4	—
Calcium chloride	CaCl_2_·2H_2_O	0.8	—
Magnesium sulfate	MgSO_4_·7H_2_O	0.6	—
Ferrous sulfate	FeSO_4_·7H_2_O	0.1	—
Manganese sulfate	MnSO_4_·H_2_O	0.016	—
Zinc sulfate	ZnSO_4_·7H_2_O	0.014	—

### Microscopy Images

#### Optical Microscopy of Microsclerotia and Mycelial Pellets

For optical microscopy, samples of either MS or P propagules were taken at 24, 48, 72, and 96 h post-inoculation of liquid media, centrifuged to recover propagules, washed twice with sterile water, and observed with a Nikon eclipse e200 optical microscope (Nikon, Japan) at 100× magnification.

#### Transmission Electron Microscopy of Microsclerotia

Two-day-old MS cultures were used for transmission electron microscopy (TEM) images following the protocol described by Huarte-Bonnet et al. (2018). Briefly, MS were washed, fixed in glutaraldehyde 2% for 2 h with soft vacuum, and washed three times with phosphate buffer. Then, MS were stained overnight with 3,3′-diaminobenzidine (DAB) (Sigma–Aldrich, USA) and washed again with the same buffer. DAB is a chemical used for determining peroxidase activity, usually employed as a peroxisome marker in microscopy images (Fahimi, 2017). Post-fixation was performed with 1% osmium tetroxide at 4°C for 1 h, followed by dehydration with a series of alcohols in a vacuum chamber. Samples were finally infiltrated with epoxy resin, and thin sections of ~70 nm were cut. Samples were observed using TEM JEM 1200 EX II (JEOL, Japan) and photographed; images were captured with an ES1000W Erlangshen CCD Camera (Gatan, USA).

### Tolerance to UV-B Radiation of Microsclerotia and Mycelial Pellets

Four-day-old propagules from cultures of both MS and P were washed and suspended in sterile water. Aliquots from each suspension containing a total of 100 propagules were inoculated onto water agar 2% (wt/vol) medium in Petri dish (80 × 10 mm) and exposed to UV-B radiation as described by Fernandes et al. (2007) and Pereira-Junior et al. (2018). The plates were irradiated at 1,283.38 mW m^−2^ of Quaite-weighted irradiance (Quaite et al., 1992) in a chamber containing 4 UV lamps (UVB-313 EL/40W; Q-Lab Corporation, Westlake, USA) for 0.5, 1, 2, 3, 4, and 5 h, which corresponded to the doses of 2.31, 4.62, 9.24, 13.86, 18.48, and 23.10 kJ m^−2^, respectively. All plates previously open were covered with a 0.13-mm-thick cellulose diacetate film (JCS Industries, La Mirada, USA). Cellulose diacetate blocks UV-C radiation (<280 nm) and short-wavelength UV-B (280–290 nm) but allows the passage of UV-B radiation (290–320 nm) and minimal UV-A (320–400 nm) emitted by the lamps. Spectral irradiance was measured with a USB 2000+ Rad spectroradiometer (Ocean Optics, Dunedin, USA). Control plates were covered with aluminum foil to block all UV radiation (Pereira-Junior et al., 2018). After UV-B exposure, the plates were incubated for 10 days at 27 ± 1°C in the dark to produce conidia from either MS or P; the conidial production and viability were then determined as described below. Three tests (two repetitions each) were conducted in different days.

### Tolerance to Heat of Microsclerotia and Mycelial Pellets

One-milliliter aliquots containing 100 propagules of either MS and P were prepared as described above into glass test tube with rubber stoppers (16 × 100 mm) and exposed to 45°C in a thermostatic bath for 0.5, 1, 2, 3, 4, and 5 h. Control tubes remained at 27°C. After each time exposure, the samples were centrifuged, and then 500 μL of supernatant was removed. The remaining volume was inoculated onto water agar 2% (wt/vol) medium in Petri dish (80 × 10 mm). After inoculation, the plates were incubated for 10 days at 27 ± 1°C in the dark to produce conidia from either MS or P propagules; the conidial production and viability were then determined as described below. Three tests (two repetitions each) were conducted in different days.

### Evaluation of Conidial Production and Viability After Exposure of Microsclerotia and Mycelial Pellets to UV-B Radiation and Heat

Conidial production and viability were assayed in MS and P propagules exposed to either UV-B radiation or heat. Conidia produced 10 days post-inoculation on water agar plates were harvested using 0.05% Tween 80 solution. By serially diluting conidial suspension, the production of conidia was quantified in each sample using a hemocytometer under optical microscope at 400× magnification. Conidial viability was assayed by inoculation of 20 μL of each propagule suspension in the center of a Petri dish (35 × 10 mm) containing 8 mL PDAY plus 0.002% (vol/wt) benomyl (50% active ingredient; Benlate®, DuPont, São Paulo, Brazil) (Braga et al., 2001) and 0.05% (vol/wt) chloramphenicol (INLab Confiança, Diadema, SP, Brazil). Plates were incubated for 48 h at 27 ± 1°C in the dark. Two drops of cotton blue were applied with a Pasteur pipette over the inoculum in each plate, and germination was immediately assessed at 400× magnification. A minimum of 300 conidia were evaluated per plate as germinated or non-germinated, and the relative percent viability of conidia was calculated according to Braga et al. (2001).

### Gene Expression Analysis

MS cultures were sampled at 24, 48, 72, and 96 h for RNA extraction and two-step real-time polymerase chain reaction (RT-PCR) analysis. Each aliquot (10 mL) was collected into screwed 15-mL centrifuge plastic tube and centrifuged for 3 min at 7,500 rpm. The supernatant was discarded, and the centrifugation pellet was washed with sterile water. The supernatant was again discarded, and the process was repeated three times. Then, MS propagules were harvested with a microbiological loop, and exposed to liquid nitrogen. Samples were ground with mortar and pestle, and 100 mg of each sample immediately transferred to 2 mL microcentrifuge tubes containing 1 mL Trizol (Invitrogen, USA). Total RNA extraction was performed according to manufacturer instructions. Total RNA samples were treated with DNase by using the Turbo DNA**-**free Kit (Ambion, USA). RT-PCR was carried out with iScript cDNA Synthesis Kit and iQSYBR Green Supermix (Bio-Rad, USA). Amplification was performed on a StepOne Plus equipment (Applied Biosystems, USA). In order to confirm that only single products were amplified, a temperature-melting step was then performed. The primer sequences used are listed in Table 2. Glyceraldehyde-3-phosphate dehydrogenase gene (*Mrgpd*) was used as housekeeping gene. Relative expression ratio of each target gene was calculated with the ΔΔCt approach, using MS harvested after 24-h growth as control. Three independent biological replicates were tested, with technical duplicates for each sample.

**Table 2:** Oligonucleotides used in this study.

**Gene (acronym used)**	**Forward (5^′^-3^′^)**	**Reverse (5^′^-3^′^)**
Peroxin 5 (*Mrpex5*)	TTTGTCCGGGCTCGCTACAATC	ATTTCGTGCGCCTTGCTTCG
Peroxin 7 (*Mrpex7*)	CCTGGCTTGGTCGGAAATCAAC	TGTTTCGCGCTTGTGTTCGTG
Peroxin 14/17 (*Mrpex14/17*)	AGGTCCAAAGGCATCAGCGAAG	TGAGCGTTGCCGAGTTGTGC
Peroxin 19 (*Mrpex19*)	ATGCCGCTCCCAAGGAATCC	TCAAACTGCTGCTGCATTTCCG
Glutathione peroxidase (*Mrgpx*)	GGGCAAAGTCGTCCTCATCGTC	TGGCCGCCAAACTGGTTACAG
Hydrophobin *(MrssgA*)	GTGTATTGCTGCAACAAAG	AGACCATTTTGCTGGACATTG
Superoxide dismutase 1 (*Mrsod1*)	CCAATGGCTGCACTTCTGCTGG	TGTGAGGGCCGATGAGCTTGAC
Superoxide dismutase 2 (*Mrsod2*)	CCAGCATCTCGGCGCAAATC	CCAGCATCTCGGCGCAAATC
Catalase A (*MrcatA*)	GTCGGCGCACAACAACTTCTG	CCAGTCGAACTTGACGACGTGC
Catalase B (*MrcatB*)	ACAGGATCAGCCACGACATCGC	TCCTTGAGAGCGTTCGCCTGAG
Catalase P (*MrcatP*)	TGCCCAATGGAGCCACAACTTC	GCAAAGGCATCGGCGAACTG
Polyketide synthase 1 (*Mrpks1*)	CATTCCGCCTCTCTCATTGCC	TGTGCGGCGCATGATATGG
Polyketide synthase 2 (*Mrpks2*)	CATCAGCGCCATCGGTTTAGAC	CGGGATAGGGATTGGTTTGTGG
Laccase 1 (*Mrlac1*)	AGGGAGACCGCACAGGATTGTG	ACTGGCTCCAATCCGACACGAC
Laccase 2 (*Mrlac2*)	TCCCTGGGTCAACGAAAGCC	CGCCGCGATAAAGTTCATGC
Laccase 3 (*Mrlac3*)	TCGGCTCAAGTGTCGTGTCCAC	CCGATCCTGTTGCCCAAACG
Glyceraldehyde-3-phosphate dehydrogenase (Mrgpd)^*^	GACTGCCCGCATTGAGAAG	AGATGGAGGAGTTGGTGTTG

* *Housekeeping gene*.

### Statistical Analyses

Statistical software R v.3.6.1 (R Core Team, 2018) was used in all analyses performed. Log2-fold data on gene expression level were fitted to a linear mixed model with normal distribution and fixed effects attributed to gene class, evaluation time, and their interaction term, and random effect was attributed to replicate to account for repeated measures over time. As interaction was significant, then means of gene expression levels were compared for each time interval and also among time intervals for each gene class. Count data on propagule yields (MS and P) were fitted to generalized linear model (GLM) with negative binomial distribution with log link function, whereas the biomass production data were fitted to a linear model (LM) with normal distribution, both models including a fixed effect for “propagule type” in the linear predictor. Similarly, LM with normal distribution was fitted to propagule size.

Regarding the UV-B tolerance assay, log_10_-transformed conidial production data were fitted to a LM with normal distribution. For heat tolerance, conidial production was fitted to a GLM with quasi-Poisson distribution and log link function. Fixed effects in both models were attributed to “propagule type,” “time of exposure,” and their interaction term in the linear predictor. Furthermore, analysis of variance (type II tests) and analysis of deviance (type II tests) were performed to assess for significance of fixed factors in these models, respectively. Pairwise mean comparisons of two samples (MS vs. P) were conducted via contrast estimates with *t* ratio test, whereas multiple pairwise comparisons of means were carried out with Tukey honestly significant difference (HSD) test, all at significance of 5%. Packages “emmeans” (Lenth, 2020), “mass” (Venables and Ripley, 2002), and “ggplot2” (Wickham, 2016) were employed in these analyses.

## Results

### Characterization of Microsclerotia and Mycelial Pellets

**Figure 1:**
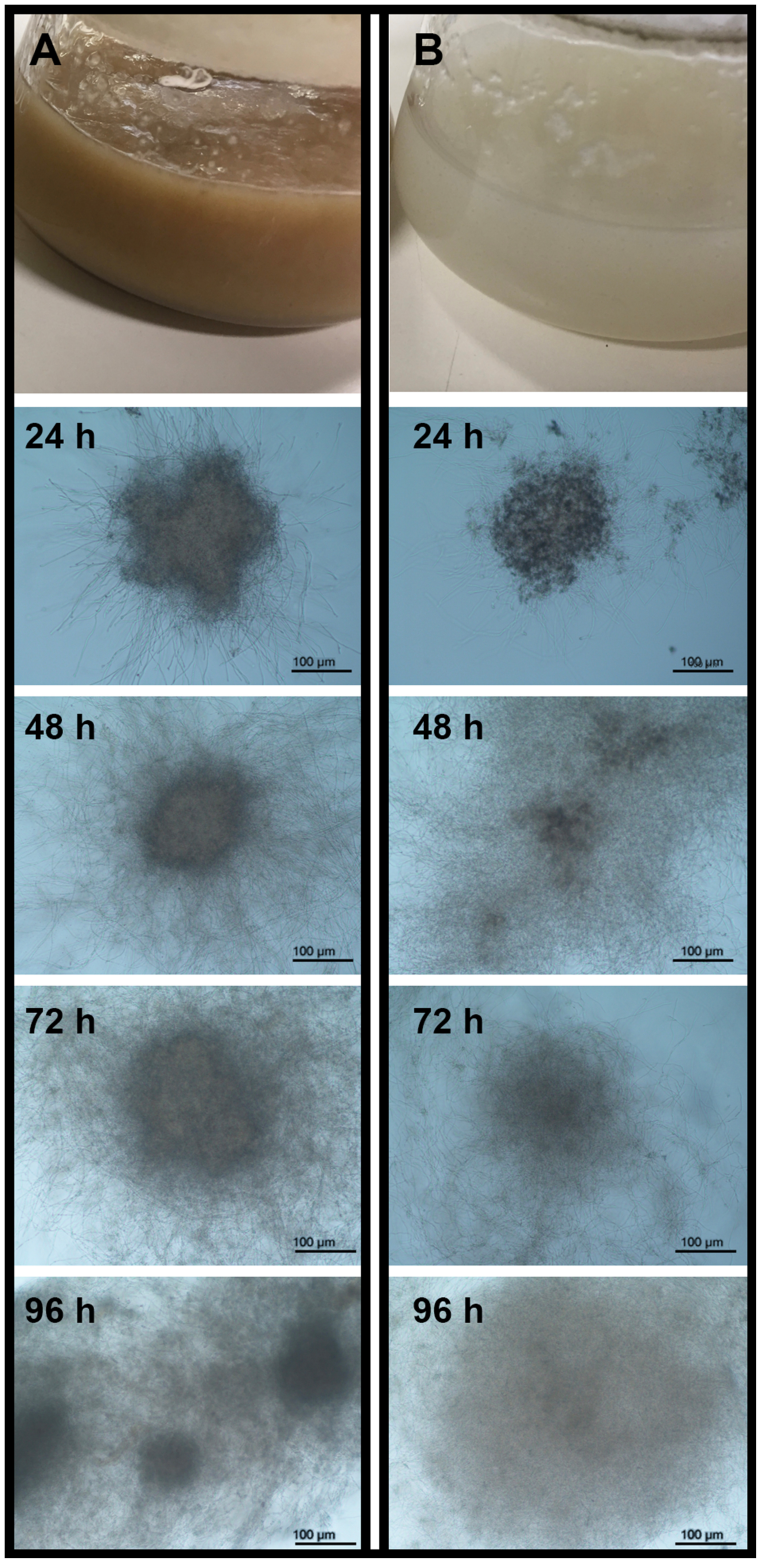
Microsclerotia **(A)** and mycelial pellets **(B)** of *Metarhizium robertsii* ARSEF 2575 produced in submerged liquid medium. From top to bottom is shown: liquid cultures in Erlenmeyer flasks at the end of fermentation (96 h), and the propagule formation registered at 100× magnification with a light microscope at 24, 48, 72, and 96 h.

**Figure 2:**
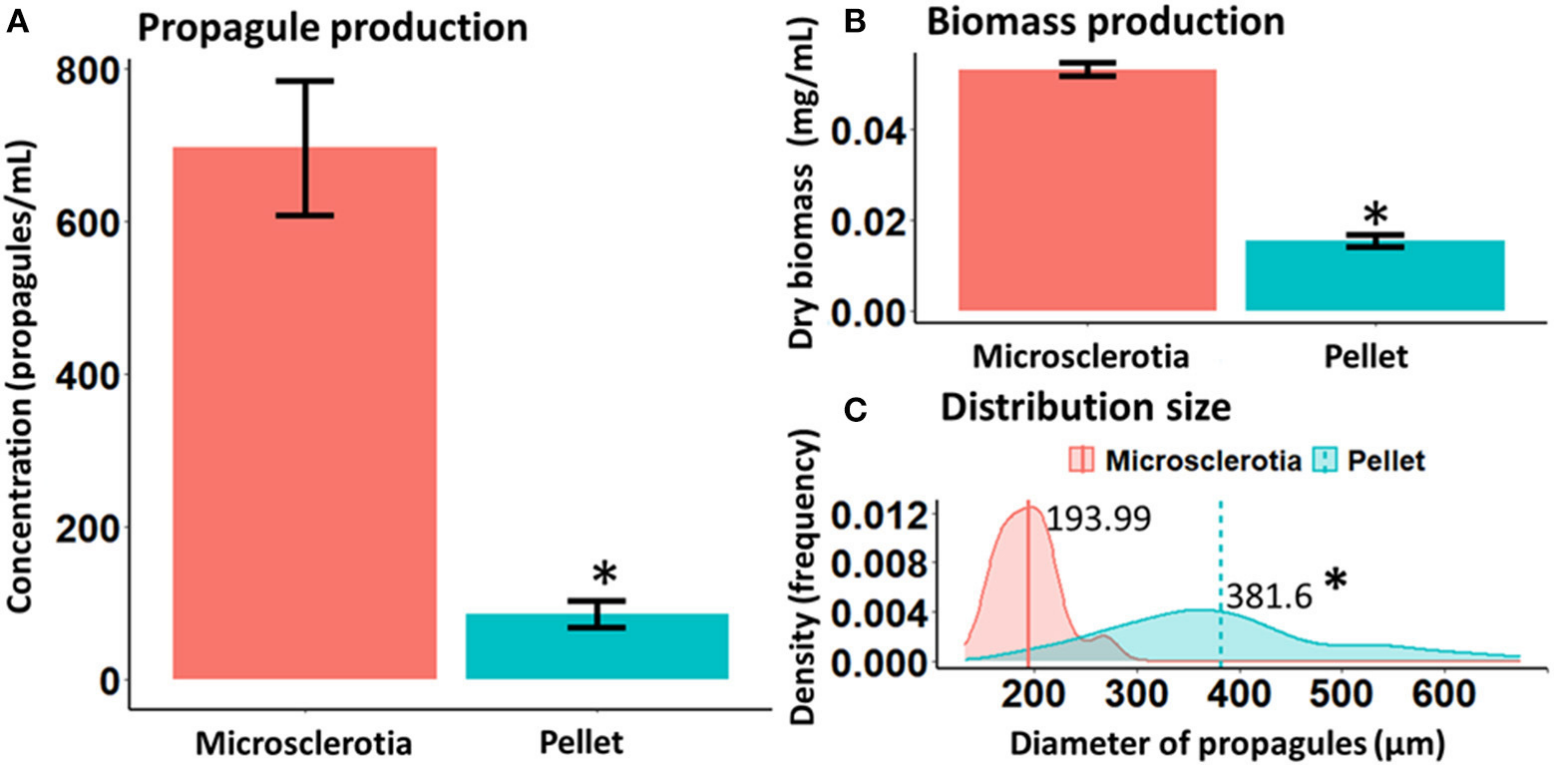
Yield **(A)**, biomass **(B)**, and distribution size **(C)** of microsclerotia and mycelial pellets obtained from 96-h-old liquid cultures of *Metarhizium robertsii* ARSEF 2575. *p < 0.05.

*M. robertsii* strain ARSEF 2575 produced either MS or P propagules in liquid cultures (Figure 1). The initial pH value of both culture media used was 5.0 and varied between 4.0 and 4.5 during fermentation. After 96-h cultivation, both propagules differed significantly on yield (MS = 700 propagules mL^−1^, *P* = 90 propagules mL^−1^) [χ^2^ = 83.64; degrees of freedom (df) = 1, 14; *p* < 0.05] and biomass production (MS = 0.053 g mL^−1^, *P* = 0.015 g mL^−1^) (*F* = 362.2; df = 1, 14; *p* < 0.05) (Figure 2). MS, but not P, showed a more compact hyphal aggregation with formation of a central medulla of thin-walled hyphae (Figure 1). Germinated conidia differentiated into MS between 6 and 18 h after inoculation, forming visible hyphal aggregates at 24 h, and compact and mature MS at 96 h with typical darker pigmentation than mycelial pellets. Optical images after 96-h growth exhibited P propagules with larger size (263–485 μm) than MS (174–226 μm); however, the latter showed higher density with more distinct curve of distribution size (*F* = 164.9; df = 1, 118; *p* < 0.0001, Figure 2).

### Tolerance to UV-B Radiation and Heat of Microsclerotia and Mycelial Pellets

**Figure 3:**
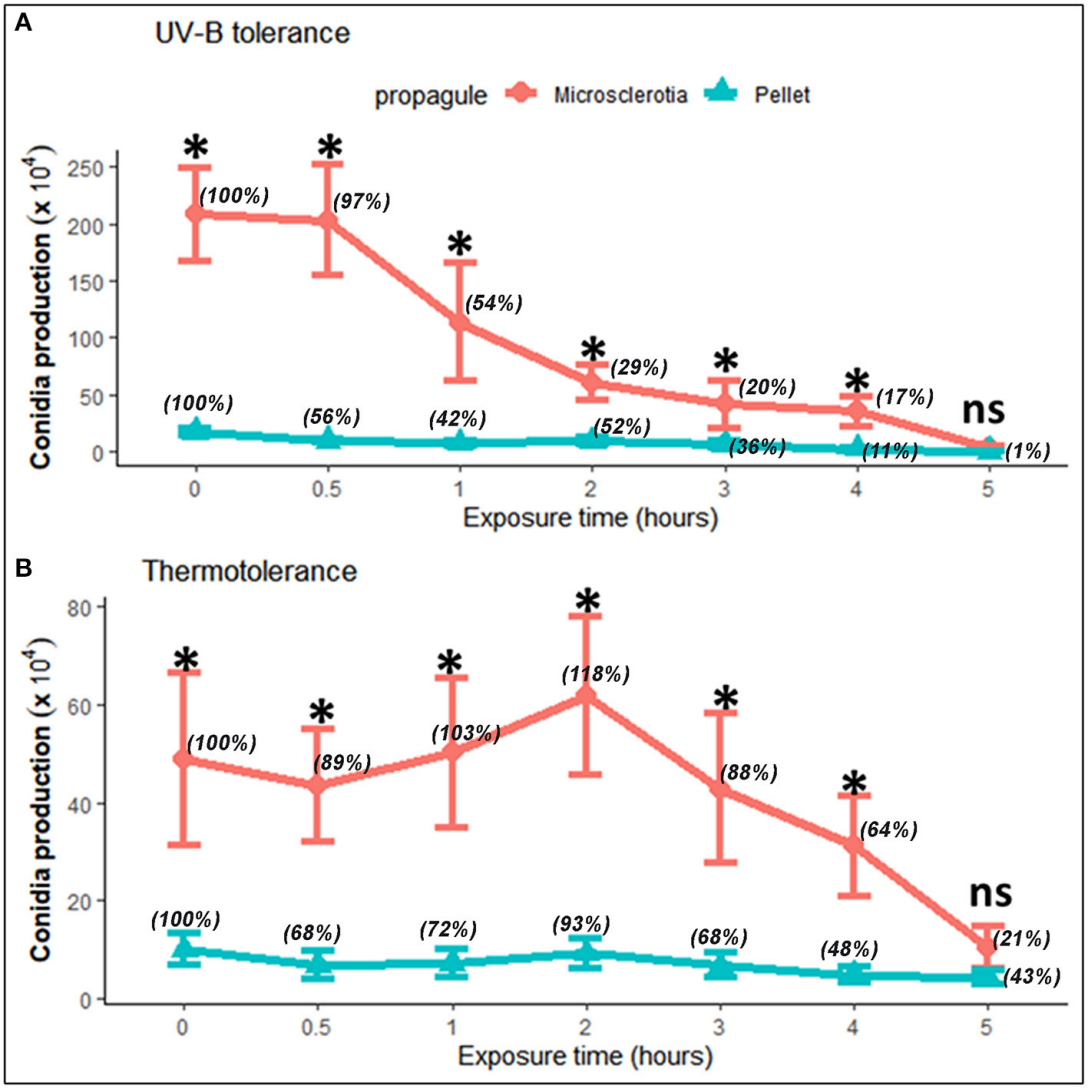
Tolerance of microsclerotia and mycelial pellets of *Metarhizium robertsii* ARSEF 2575 to artificial UV-B radiation [1,283.38 mW m^−2^ of Quaite-weighted irradiance (Quaite et al., 1992) for 0 (control), 0.5, 1, 2, 3, 4, and 5 h, which corresponded to the doses of 0 (control), 2.31, 4.62, 9.24, 13.86, 18.48, and 23.10 kJ m^−2^, respectively] **(A)**, and heat (45°C) **(B)**. Values indicate mean and standard error. Relative percentage values for each type of propagule are shown in parentheses. At each exposure time, the significant differences between propagules are shown with an asterisk (*p* < 0.05). ns = not significant.

Fungi exposed to UV-B radiation (1,283.38 mW m^−2^) had their conidial production decreased in a time-dependent manner; however, both MS and P resulted to be tolerant after exposure to heat (45°C). Comparing both types of propagules, conidial productivity of MS was higher than those of P even without treatment by UV-B or heat at all exposition times assayed, except after 5-h exposure (*F* = 12.38; df = 6, 70; *p* < 0.0001; and *F* = 3.24; df = 6, 70; *p* = 0.007, respectively) (Figure 3). The effect of UV-B radiation and heat on MS sporogenesis was evident resulting in significant reduction on conidial production after 2- and 4-h exposures, respectively, in relation to its initial production from unexposed MS (Figure 3). Overall, the susceptibility of P was considerably higher than MS after exposure to UV-B (*F* = 52.78; df = 1, 70; *p* < 0.0001) and heat stress (*F* = 74.74; df = 1, 70; *p* < 0.0001). The mean viability of conidia produced from UV-B- or heat-stressed MS or P was higher than 97% in each test, regardless of the exposure time.

### Ultrastructural and Gene Expression Analyses During Microsclerotial Differentiation

**Figure 4:**
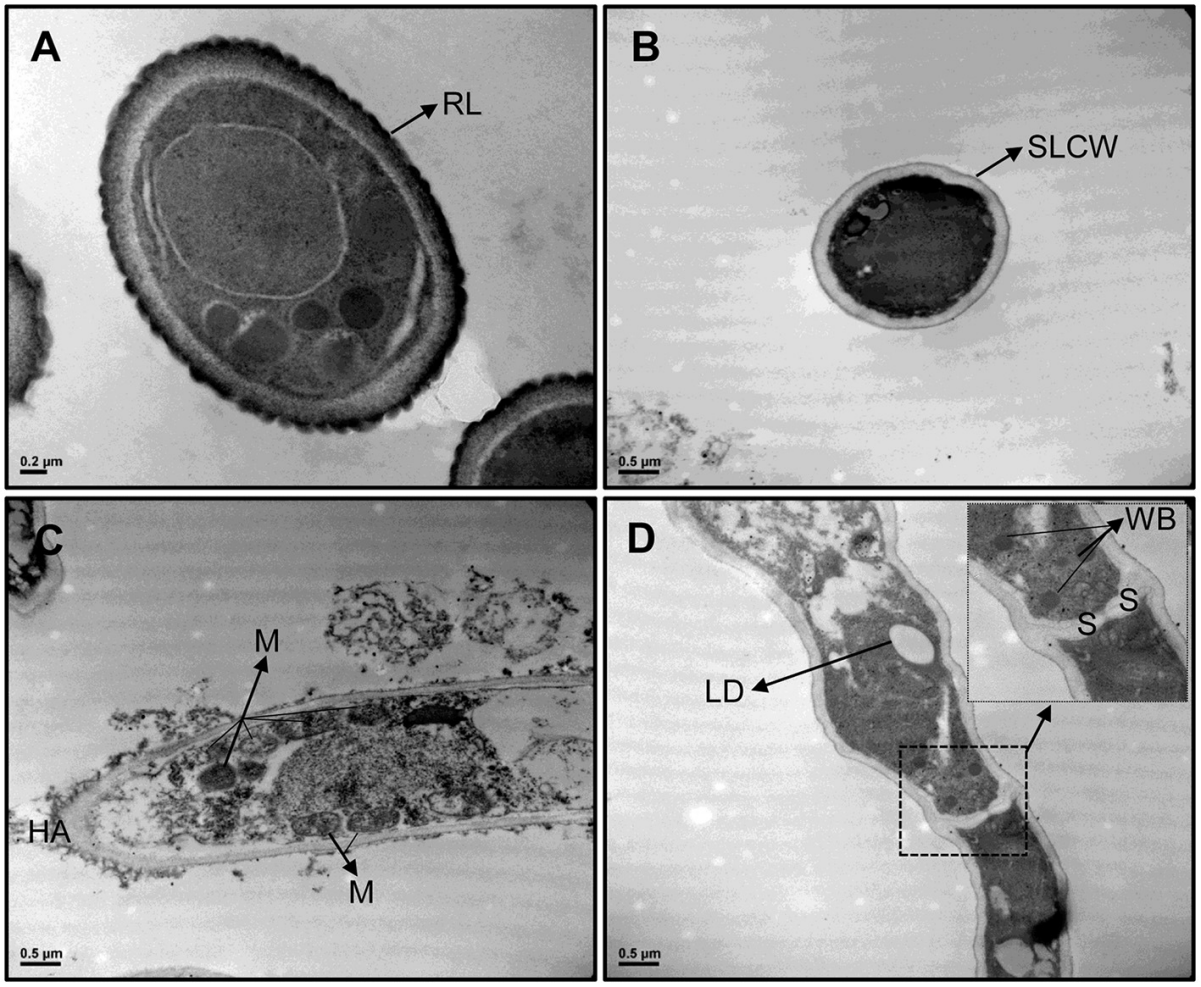
TEM images from *Metarhizium robertsii* ARSEF 2575 conidia **(A)** and microsclerotial propagules **(B–D)** at 30,000× magnification stained with DAB. Cross section of both propagules showed differences in cell wall and ultrastructure morphology. Because of DAB reaction, microsclerotia exhibited black dots and black areas inside the cells, in hyphal apex, and in cell interconnections. Several mitochondria, peroxisomes, and Woronin bodies are visible and strongly stained in microsclerotia, but not in conidia. RL, rodlet layer; SLCW, single layered cell wall; HA, hyphal apex; M, mitochondrium; LD, lipid droplet; WB, Woronin body; S, septum.

TEM images revealed differences in cell wall configuration between conidia (rodlet layer is observed) and MS (single-layered cell wall is observed) (Figure 4). The organelles observed in MS comprised several mitochondria, lipid droplets, and peroxisomes located next to hyphal septa, suggesting Woronin bodies (WBs), a peroxisome-derived, dense core microbody with a unit membrane found near the septae that divide hyphal compartments in filamentous Ascomycota. Also, high peroxidase activity was detected in MS but not in conidia. Peroxidase activity was demonstrated as small black dots due to DAB reaction with H_2_O_2_ inside the cells and also in hyphal apex (Figure 4).

**Figure 5:**
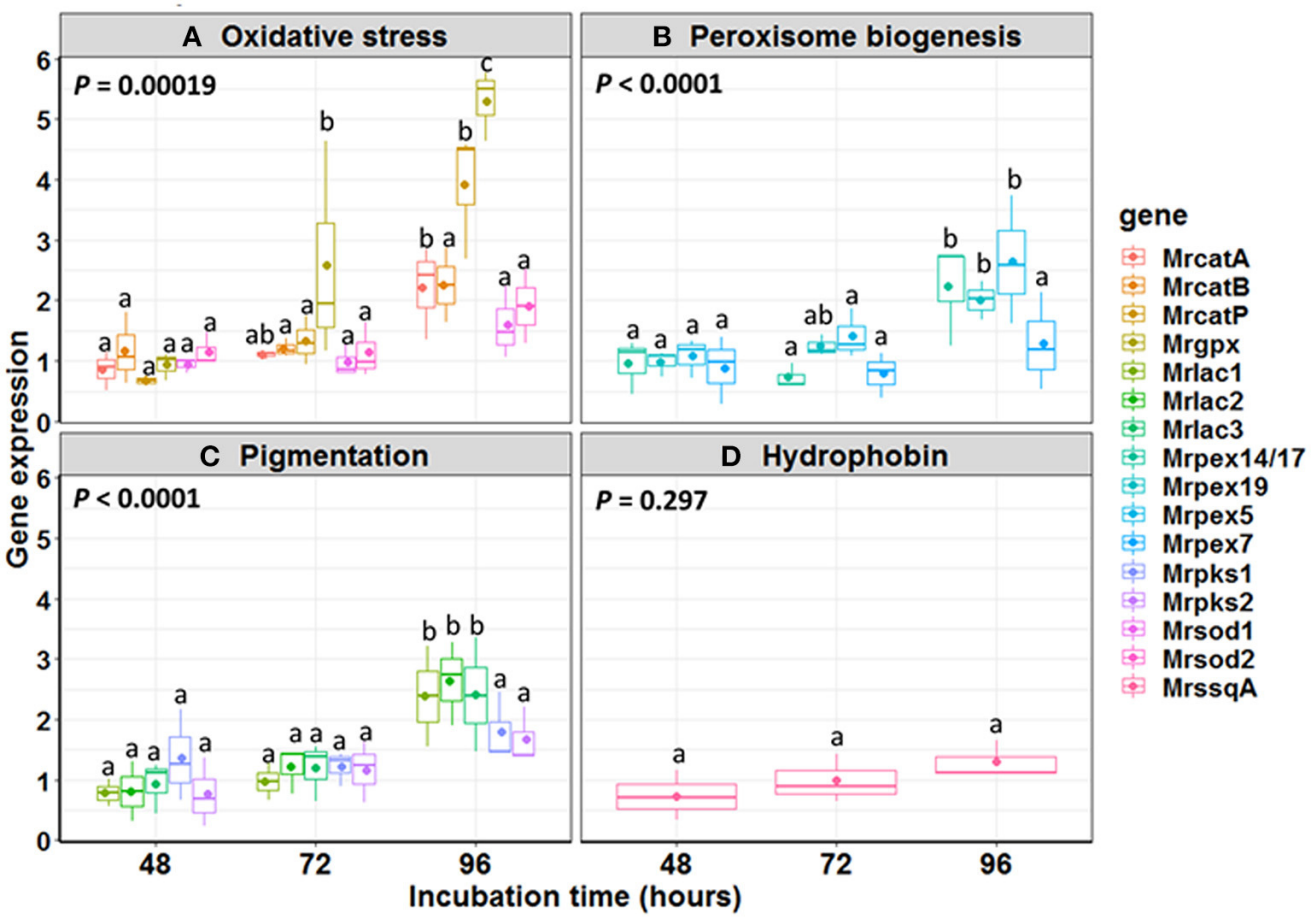
Relative expression ratio of genes involved in **(A)** oxidative stress, **(B)** peroxisome biogenesis, **(C)** pigmentation, and **(D)** hydrophobin during *Metarhizium robertsii* microsclerotial differentiation. The boxes include 25–75% of the values; the line inside the boxes is the median (50%) of the distribution of all values, and the vertical bars represent the non-outlier range. Mean values are indicated by dots inside each boxplot. Mean comparisons were made between time intervals within each gene class, and different letters highlight significant differences between the gene expression. Significance level used: α = 0.05 according to Tukey HSD test.

The expression pattern of 16 genes putatively involved in MS formation was analyzed (Figure 5). Based on previous studies (Huarte-Bonnet et al., 2018, 2019, 2020; Pereira-Junior et al., 2018), the selected genes were those associated with oxidative stress (*MrcatA, MrcatB, MrcatP, Mrsod1, Mrsod2, Mrgpx*), peroxisome biogenesis (*Mrpex5, Mrpex7, Mrpex14/17, Mrpex19*), pigmentation (*Mrpks1, Mrpks2, Mrlac1, Mrlac2, Mrlac3*), and hydrophobin rodlet layer (*MrssgA*). For each gene, the expression was measured at 48, 72, and 96 h after start of fermentation and was normalized with values measured at 24 h (control). For CAT family, *MrcatA* and *MrcatP* expression at 96 h post-inoculation was significantly higher (2.2- and 3.9-fold increase, respectively) than those found at both 48 and 72 h. The most expressed gene at 96 h was *Mrgpx* (5.3-fold induction), significantly higher (*p* < 0.01*)* than the previous point at 72 h (2.6-fold induction) (Figure 5). No induction over time was observed for SOD family. Within the peroxin family, *Mrpex14/17* (1.5-fold induction) and *Mrpex5* (2.6-fold induction) were observed at 96 h, the same as for the pigmentation-associated genes *Mrlac1* (2.4-fold induction), *Mrlac2* (2.6-fold induction), and *Mrlac3* (2.4-fold induction) (*p* < 0.001).

## Discussion

Nutritional conditions in filamentous fungal fermentation strongly influence growth and morphology of the resulting propagules (Cox and Thomas, 1992), as well as pH and aeration rate (Nair et al., 2016; Veiter et al., 2018). Fermentation conditions used in this study were favorable to produce either MS or mycelial pellets (P) from *M. robertsii* ARSEF 2575. MS development resumed with hyphal aggregates at 18 h post-inoculation, with compact structures appearing at 24 h, and mature pigmented MS propagules at 96 h. This process bears out with MS definition in entomopathogenic fungi, which consists in the formation of dark pigmented and compact hyphal threads with or without a distinct core (Jaronski and Jackson, 2008; Jackson and Jaronski, 2009; Behle et al., 2013; Mascarin et al., 2014; Goble et al., 2016; Song et al., 2016a,b; Xin et al., 2020). Pellet (P) formation was obtained in a liquid culture medium considered poor by its composition, consisting in a basal medium devoid of salts but constituted of three elements that provided the source of carbon and nitrogen required for propagule development. Some parameters, such as pH, oxygen level, temperature, and agitation speed, are very important for the growth and maturation of these propagules (Zhang and Zhang, 2016). The pH of the culture medium used during MS production in *Metarhizium* species was shown to be initially acid (pH 5.5) (Jackson and Jaronski, 2012; Behle et al., 2013; Song et al., 2015, 2017). In concordance with reports from *Metarhizium* (formerly *Nomuraea*) *rileyi* (Song et al., 2015), we found that pH dropped to 4.0 during MS development, likewise during P formation. The pH is the driving factor for electrostatic and hydrophobic interactions on specific aggregation and pellet morphology (Zhang and Zhang, 2016; Veiter et al., 2018). The medium composition was determinant for P formation with bigger diameter than MS. However, even at the same agitation speed and temperature conditions, we found higher biomass accumulation and propagules concentration in MS than in P. This result corroborates with studies in *Metarhizium anisopliae*, which previously demonstrated that liquid cultures with carbon-rich media accumulated more biomass and yielded more MS numbers (Jackson and Jaronski, 2009), while fermentation media with higher nitrogen concentrations also resulted in greater biomass accumulation and MS yields than those grown in nitrogen-deficient medium (Behle and Jackson, 2014). Nutritional and environmental growth conditions play an essential role in fungal development by providing energy source and cofactors for biochemical reactions, and these factors also exert a remarkable impact on the formation and quality attributes of a variety of propagules employed for different purposes in pest control (Jaronski and Mascarin, 2016). For *Metarhizium* strains, reduced culture viscosity during the MS formation in a nutrient-poor medium was observed, because of rapid exhaustion of nutrients (Mascarin et al., 2014). Accordingly, we found that the culture medium for MS production appeared more liquid and less thick than the culture medium for P production. Lower viscosity is also associated with higher oxygen supply in fungal submerged liquid cultures, which can enhance fungal growth and specific propagule yields, as reported before (Mascarin et al., 2015).

In previous studies using other fungal propagules such as blastospores or aerial conidia, the effects of abiotic factors were addressed to assess tolerance and survival of some fungi commonly used in biological control (Braga et al., 2001, 2015; Paixão et al., 2017; Pereira-Junior et al., 2018; Bernardo et al., 2020). Conidia from *Metarhizium* species exposed to UV-B radiation (irradiances between 920 and 1,200 mW m^−2^) had both germination and survival affected in a dose- and time-dependent manner (Braga et al., 2001). Conidia from the same strain used in this study (ARSEF 2575) exposed to 866.7 mW m^−2^ Quaite-weighted irradiance showed a certain degree of UV-B tolerance in relation to the dose used (3.9–6.2 kJ m^−2^) (Pereira-Junior et al., 2018). Conidia and blastospores from *M. robertsii* IP 146 were equally tolerant to UV-B radiation (743.7 mW m^−2^ irradiance); however, the relative viability was lower than in aerial conidia when exposed to 45°C (Bernardo et al., 2020). Recently, the viability of MS exposed to UV-B radiation (4.0 kJ m^−2^) varied greatly among *Metarhizium* spp. isolates, from very susceptible to quite tolerant ones (Corval et al., 2021). In the present study, we demonstrated for the first time the comparative tolerance of MS and P from *M. robertsii* ARSEF 2575 to UV-B radiation (1,283.38 mW m^−2^). Although the effects of either UV-B or heat exposure did not inhibit conidial production (sporogenesis) by MS or P post-inoculation on water agar medium, we found that MS possess the ability to withstand better both stressful conditions as shown by their higher conidial production than P. Furthermore, we can infer that based on the number of conidia produced, the heat stress scenario (45°C) has a stronger detrimental effect on both MS and P propagules than UV-B radiation. Conidial production was evaluated after 10 days of incubation, and such period of time was necessary to promote complete sporulation in MS and P propagules under a highly humid microenvironment generated by the water agar medium used as an artificial substrate.

TEM microscopy revealed differences between conidia and MS in some intracellular structures such as cell wall, peroxisomes, and WBs, as well as peroxidase activity. WBs are organelles found exclusively in mycelium of filamentous fungi, and their function is sealing the septal pore in response to injury, allowing the rest of the mycelium to continue growth and to confer stress resistance, among other proposed functions (Liu et al., 2008). This organelle is, in fact, a special type of peroxisome found at the cell periphery or in association with the septum (Jedd and Chua, 2000; Liu et al., 2011). Thus, two types of peroxisomes can be found in filamentous fungi; one type is immobile sealing pores between hyphal cells, and the other is mobile and actively inserted into growing hyphae (Knoblach and Rachubinski, 2016). Both types are associated with anabolic and catabolic pathways, peroxide metabolism, oxidation of fatty acids, and the biosynthesis of phospholipids (Jedd and Chua, 2000). Recently, the protein forming hexagonal crystals inside WBs (HEX1) was functionally characterized in the mycelium of *M. robertsii*; MrHex1 was responsible for WB formation and involved in sealing septal pores, but unexpectedly, it does not seem to have any function regarding stress tolerance and virulence (Tang et al., 2020). For the first time, we noted the presence of peroxisomes in *M. robertsii* MS by TEM after DAB fixation, indicating high peroxidase activity inside cells and in-cell interconnections during MS formation. Most peroxisomes were found near the septa of hyphae, suggesting that they might be, in fact, WB. Peroxisomes and peroxidase activity were studied on mycelial pellets and MS-like pellets of *B. bassiana* (Huarte-Bonnet et al., 2018, 2019). Both studies, in accordance with this study on *M. robertsii*, also reported induction of *pex* genes encoding proteins named peroxins (PEXs). In general, PEXs are associated with peroxisomes biogenesis, and most of them are involved in the transport of matrix proteins from the cytosol into the peroxisome lumen (Kiel et al., 2006; Opaliński et al., 2010; Pieuchot and Jedd, 2012). The characterization of *pex* genes was done in other filamentous fungi such as *Magnaporthe oryzae* (*MoPex7*, expressed during short-chain fatty acid metabolism and pathogenesis) (Goh et al., 2011) and *Fusarium graminearum* (*FgPex4*, involved in regulation of hyphal growth, sexual and asexual reproduction, virulence, cell wall integrity, and elimination of ROS) (Zhang et al., 2019). The gene family *Pex14/17* (also known as *Pex33*) has been identified as fungal-specific gene encoding peroxin associated with conidiospore formation and peroxisome biogenesis and encoding a protein located in peroxisomal membrane (Managadze et al., 2010; Opaliński et al., 2010). Some orthologs of *Pex14/17* have been also characterized in *M. oryzae*, associated with conidial germination, germ tube elongation, and initial emergence of appressoria and as docking receptor peroxisomal membrane protein (Li et al., 2017). In *B. bassiana, Bbpex14/17* but also *Bbpex5, Bbpex7*, and *Bbpex19* have been shown to be induced during formation of MS-like pellets (Huarte-Bonnet et al., 2019). In this study, the genes *Mrpex5* and *Mrpex14/17* were the only upregulated at 96 h post-inoculation in liquid medium. Although in eukaryotic cells both *Pex5* and *Pex7* have been reported as cycling cytosolic receptors recognizing the peroxisomal targeting signals PTS1 and PTS2, respectively (Pieuchot and Jedd, 2012); in *Neurospora crassa*, the docking complex of peroxisomal matrix protein import is composed of PEX14/17 (PEX33) interacting with itself and with the PTS1-receptor PEX5 (Managadze et al., 2010). This result suggests that both genes might be expressing and acting together in this function during the development and aging of *M. robertsii* MS.

We also found an oxidative stress scenario during MS differentiation. ROS production has been reported during cell differentiation in fungi and specifically for sclerotia differentiation (Georgiou et al., 2006). Studies on the hyphal aggregation processes indicate they are mediated by reduced oxygen entrance inside the cell due to a decreased surface-volume ratio and thus might be a mechanism of fungal mycelial adaptation to an increment of ROS in the microenvironment (Gessler et al., 2007). Antioxidant enzymes such as SODs, CATs, and GPxs act as the first line of cell defense against ROS (Aguirre et al., 2005; Song et al., 2013; Liu et al., 2018; Huarte-Bonnet et al., 2019). SODs decompose superoxide anion and singlet oxygen into oxygen and H_2_O_2_, and depending on the cofactors used, this family comprises three types of isoforms, i.e., Cu^2+^/Zu^2+^, Mn^2+^, and Fe^2+^ (Culotta et al., 2006). In structure and phylogeny, SODs from *M. robertsii* are homologous to those from *B. bassiana* and other filamentous fungi, but some of them are functionally distinct (Zhu et al., 2018). SOD gene expression was reported during differentiation of microesclerotia-like pellets in *B. bassiana* (Huarte-Bonnet et al., 2019) and MS development in *M. rileyi* (Song et al., 2013). Nevertheless, it was found that both *Mrsod1* (Cu^2+^/Zu^2+^ SOD) and *Mrsod2* (Mn^2+^SOD) were not induced during MS formation. Fungal CATs act during germination and growth in response to oxidative stress (Michán et al., 2002; Pedrini et al., 2006; Wang et al., 2013; Song et al., 2018; Huarte-Bonnet et al., 2019). In this study, we measured the expression pattern of two cytosolic CATs (*cat*A and *cat*B) and one peroxisomal CAT (*cat*P) (Wang et al., 2013). Only *MrcatP* was induced in mature MS, in agreement with the previous result reported on mycelial pellets and MS-like pellets of *B. bassiana* (Huarte-Bonnet et al., 2018, 2019). A peroxisomal CAT gene was also expressed during vegetative growth and sclerotial developmental stages of *Sclerotinia sclerotiorum* (Yarden et al., 2014). Interestingly, the most expressed gene from oxidative stress response was *Mrgpx*, which was induced in MS at 72 and 96 h. GPxs are known to reduce either H_2_O_2_ or organic hydroperoxides to water or their corresponding alcohols using reduced glutathione and glutathione disulfide (Margis et al., 2008; Huarte-Bonnet et al., 2015). As GPxs and CAT used both H_2_O_2_ as substrate, it might occur that H_2_O_2_ can be reduced by induction of one of the two detoxification systems. In *B. bassiana* mycelial and MS-like pellets, CATs (*BbcatA-C* and *BbcatP*) were strongly induced, but *Bbgpx* was low or not induced (Huarte-Bonnet et al., 2018, 2019), just as the opposite we found in this study for *M. robertsii* MS. Taken together, our values of *Mrgpx, MrcatP*, and *Mrsod1* expression measured at 96 h showed similarities with the study by Michiels et al. (1994), reporting that Gpx has a high protective behavior, CAT has an intermediate behavior, and Cu/Zn-SOD has a very small protective effect to protect cells against free radicals.

Hydrophobins are surface active proteins produced by filamentous fungi. They are proteins involved in the growth and morphogenetic processes (Wösten, 2001), and in *M. anisopliae* s.l., the hydrophobin-encoding gene *ssgA* was linked with appressorium development (St. Leger et al., 1992). Although earlier studies reported hydrophobin genes playing important roles in MS development of *Verticillium dahliae* (Klimes and Dobinson, 2006) and pellet formation via hydrophobic interactions in filamentous fungi (Zhang and Zhang, 2016), we have not found induction for *MrssgA* in any of the fermentation timepoints during MS development. Regarding the identification of potential pigmentation mechanisms, laccases (LACs) and polyketide synthases (PKSs) have been linked with fungal pigmentation. LACs are multicopper oxidases that catalyze the transformation of aromatic and non-aromatic compounds with the reduction of molecular oxygen to water. There are various isoforms due to the diverse physiological functions during the fungal life cycle (Rivera-Hoyos et al., 2013). In *Metarhizium* spp., *M. anisopliae* LAC (*Mlac1*) is expressed during isotropic growth and has been proposed to provide tolerance to abiotic stress during conidial pigmentation (Fang et al., 2010). *M. robertsii* and *M. acridum* LACs (*lac1, lac2*, and *lac3*) were upregulated when exposed to UV-B irradiation (Pereira-Junior et al., 2018). In this study, all three *Mrlac1, Mrlac2*, and *Mrlac3* were also induced in mature MS. On the other hand, PKS is an enzyme family implicated in the biosynthesis of polyketides with several biological activities, including pigment and biosynthesis of mycotoxins. *Pks1* and *Pks2* have been reported to be expressed in *Metarhizium* spp. *Pks1* is involved in conidial pigmentation and tolerance to environmental stresses, and *Pks2* is related to pathogenicity (Pereira-Junior et al., 2018; Zeng et al., 2018). In *M. rileyi*, the expression pattern of *pks* has been increased between initial stage and mature MS (Song et al., 2013); however, in this study, neither *Mrpks1* nor *Mrpks2* genes were induced during MS formation. As hyphal aggregation process is accompanied by the biosynthesis of pigment molecules in the mycelium (Gessler et al., 2007), and all LACs but not *pks* genes were induced at 96 h; thus, we hypothesize that the LAC pathway might be responsible for MS pigmentation. However, additional functional studies are needed to confirm this hypothesis. As a conclusion, looking at the expression pattern of genes involved in oxidative stress response, peroxisome biogenesis, and pigmentation, the upregulation of all of them was documented only at 96 h, that is, in mature MS.

In summary, *M. robertsii* is able to produce different propagules under different fermentation conditions, i.e., favoring the development of either MS or P. Despite their size twice smaller, MS exhibited higher dry biomass and concentration than P. The microsclerotial differentiation process includes at least a mechanism triggering oxidative stress, high peroxidase activity, and active peroxisome biogenesis. We propose that because of its tolerance to desiccation, heat, and UV-B, MS of this isolate could be an excellent candidate to be used in biological control of pests under environmental tropical and subtropical conditions.

## Data Availability Statement

The original contributions presented in the study are included in the article/supplementary materials, further inquiries can be directed to the corresponding author/s.

## Author Contributions

ÉF and NP conceived the project. FP, ÉF, CH-B, GM, and NP designed the experiments. FP, CR-S, and CH-B performed the experiments. FP, GM, and NP analyzed the data. FP and NP wrote the manuscript. All authors read and approved the final manuscript.

## Conflict of Interest

The authors declare that the research was conducted in the absence of any commercial or financial relationships that could be construed as a potential conflict of interest.
